# Rapid clonal analysis of recurrent tuberculosis by direct MIRU-VNTR typing on stored isolates

**DOI:** 10.1186/1471-2180-7-73

**Published:** 2007-07-30

**Authors:** Ana Martín, Marta Herránz, María Jesús Ruiz Serrano, Emilio Bouza, Darío García de Viedma

**Affiliations:** 1 Servicio de Microbiología y Enfermedades Infecciosas. Hospital General Universitario Gregorio Marañón, CIBER Enfermedades Respiratorias (CIBERES), Universidad Complutense, Madrid, Spain

## Abstract

**Background:**

The application of molecular tools to the analysis of tuberculosis has revealed examples of clonal complexity, such as exogenous reinfection, coinfection, microevolution or compartmentalization. The detection of clonal heterogeneity by standard genotyping approaches is laborious and often requires expertise. This restricts the rapid availability of *Mycobacterium tuberculosis *(MTB) genotypes for clinical or therapeutic decision-making. A new PCR-based technique, MIRU-VNTR, has made it possible to genotype MTB in a time frame close to real-time fingerprinting. Our purpose was to evaluate the capacity of this technique to provide clinicians with a rapid discrimination between reactivation and exogenous reinfection and whether MIRU-VNTR makes it possible to obtain data directly from stored MTB isolates from recurrent episodes.

**Results:**

We detected differences, between the MIRUtypes of recurrent isolates in 38.5% (5/13) of the cases studied. These included cases of i) exogenous reinfection, often with more resistant strains, ii) likely examples of microevolution, leading to the appearance of new clonal variants and iii) a combination of microevolution, coinfection and competition.

**Conclusion:**

MIRU-VNTR rapidly obtained clinically useful genotyping data in a challenging situation, directly from stored MTB isolates without subculturing them or purifying their DNA. Our results also mean that MIRU-VNTR could be applied for easy, rapid and affordable massive screening of collections of stored MTB isolates, which could establish the real dimension of clonal heterogeneity in MTB infection.

## Background

Until recently, infection by *Mycobacterium tuberculosis *(MTB) has been assumed to be clonally simple, and a tuberculosis (TB) case was traditionally thought to be infected by a single MTB strain. The introduction of molecular biology tools into the clinical microbiology laboratory has shown that this infection is sometimes clonally complex. In this sense, fingerprinting of clinical cultures has revealed i) exogenous reinfection as a cause of recurrences more often than originally thought [1-5], ii) simultaneous coinfection with different MTB strains [4-7] iii) microevolution phenomena [8,9] and iv) compartmentalization of the infection, with different strains infecting different tissues [9,10], or even independent lung sites [11].

Clonal complexity in TB is now well documented, and clinicians are increasingly requesting characterization of the strains involved in these cases, mainly to discriminate between reinfection and reactivation. However, RFLP-based or spoligotyping-based fingerprinting approaches do not provide results quickly enough to include MTB genotypes in the pool of the first-line microbiological data available to take clinical, therapeutic or epidemiological decisions.

A new PCR-based fingerprinting technique, MIRU-VNTR [12], has been considered a suitable alternative for the simple and rapid detection of clonal complexity in TB [7,13,14]. We verified whether MIRU-VNTR could provide a rapid answer in conditions mimicking a real clinical situation: when MTB is cultured from a patient who had a previous TB episode and the clinician demands the discrimination between reactivation and exogenous reinfection. We evaluated the efficiency of MIRU-VNTR for the rapid analysis of clonal complexity in recurrent cases directly from a collection of frozen-stored MTB isolates, without subculturing them or purifying DNA.

## Results

We were able to analyze the sequential MTB isolates of 58 patients with two or more recurrent TB episodes (2–5 episodes separated by intervals of between 6 months and 5 years). Thirty-two stored MTB isolates from 13 representative cases of recurrences (Table 1) were selected for MIRU-VNTR genotyping.

**Table 1:** Characteristics of the patients. IVDU: intravenous drug user

PATIENT	SEX	AGE	RISK FACTORS	EPISODES*	EVIDENCE OF NON-ADHERENCE TO THERAPY	CLONAL HETEROGENEITY
A	Female	44		9 (4)	+	+
B	Male	50	IVDU	2	+	
C	Male	46		4 (2)		
D	Male	45	HIV+, IVDU, Prison	2	+	
E	Male	28		3	+	
F	Male	57	Alcoholism	4 (3)		
G	Male	52	Alcoholism	2	+	
H	Male	32	HIV+, IVDU	4	+	
I	Female	31	HIV+, IVDU	2		+
J	Male	46	HIV+, IVDU	2		+
K	Male	46	HIV+, Alcoholism	2		+
L	Male	53	IVDU, Alcoholism, Prison	3 (2)		+
M	Male	53		2		

*: The number of episodes with MTB isolates that could be analyzed by MIRU-VNTR are indicated in brackets.

We could amplify at least ten of the 12 MIRU targets from twenty-nine stored clinical isolates (90.6%). In an additional case we were only able to obtain a fingerprint from six loci. For the remaining two isolates, no MIRU loci were amplified (corresponding to two isolates from the years 1997 and 2003) and for one of these, MTB was not recovered after subculture.

### Clonal analysis of recurrences

**Figure 1:**
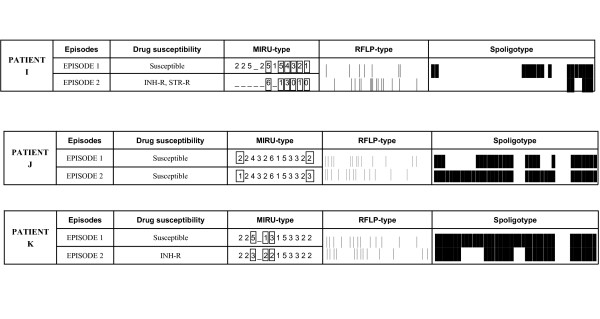
Susceptibility and genotyping features of the isolates from cases with reinfections. Differences in the MIRUtypes are boxed

**Figure 2:**
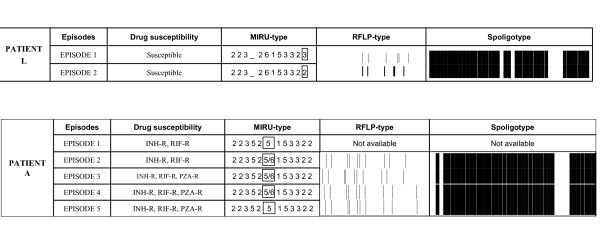
Susceptibility and genotyping features from cases with subtle changes in the clonal composition of the isolates. Differences in the MIRUtypes are boxed

We compared the genotypes of the recurrent isolates obtained directly from the stored isolates. We found different MIRU types in the recurrent isolates in 5 of the 13 patients (38.5%; cases A, I, J, K, L) and identical MIRU types were detected for the remaining eight cases (Table 1, Figures 1 and 2). A highly discriminatory MIRU set of 15 loci was also applied with these eight cases and identical patterns for the sequential episodes were again obtained (data not shown). In three of the patients with clonal differences the isolates differed in two or more loci, including one case whose sequential isolates differed in six loci.

For patients I, J, K, i.e. those with differences in two or more than two MIRU loci, the RFLP and spoligotyping analysis confirmed the participation of different MTB strains (reinfection) in the first and recurrent episodes (Figure 1). In addition, in two of these cases (I and K) the susceptibility pattern of the sequential isolates changed from susceptible to resistant (Figure 1).

In patients L and A, the differences between recurrent isolates were more subtle, and affected only one locus (Figure 2). RFLP and spoligotype showed identical patterns for these clonal variants. Therefore we considered that these cases could be microevolution phenomena and not true reinfections. Whereas case L was an example of likely simple microevolution, a much more complex situation was detected for case A (Figure 2). This patient was initially infected by a single strain. However, a clonal variant appeared in the second episode, and continued to coinfect the patient together with the parental clone. This coinfection by both clonal variants was detected in the second, third and fourth episodes and finally, the first variant was the only one recovered from the fifth episode. In this case we observed differences in susceptibility patterns for pyrazinamide in the sequential episodes (Figure 2).

## Discussion

The introduction of molecular biology tools in the clinical setting has revealed that MTB infection is clonally complex and that exogenous reinfection [1-5], simultaneous coinfection [4-7], compartmentalization [9,10] and microevolution [8,9] are examples of this clonal complexity.

In the past, clinicians assumed that clonal complexity had no impact on the management of TB and it was considered to be merely a refinement of the microbiological analysis. However, there are reports of TB cases with clonal complexity in which the strains can differ in their susceptibility patterns [11,15-17], infectivity [18], or ability to infect extrarespiratory sites [9,10]. This has alerted clinicians to the usefulness of accessing to advanced molecular microbiological analysis.

The procedures usually applied to detect clonal heterogeneity are based on low- intensity RFLP bands or on the analysis of multiple single MTB colonies from culture. They are cumbersome, require expertise and cannot provide a quick answer in a real clinical setting. Recently, a novel genotyping method, MIRU-VNTR [12], has been proposed as an alternative to simplify and optimize the detection of clonal complexity directly from culture [13,19].

In this study, we evaluate the efficiency of MIRU-VNTR at providing a rapid answer to a frequent request by clinicians, the discrimination between reactivation and exogenous reinfection in recurrent cases. Recurrence is not an anecdotal aspect of TB, and between 1 and 11% of cases have a second recurrent episode (7% in our context). Rapid identification of recurrences caused by exogenous reinfection could influence therapeutic and epidemiological decisions because susceptibility could be different and the patient should be considered a new case. Besides, when a case is assumed to be a relapse, rapid information on exogenous reinfection by a strain spreading in the community could indicate new recent-transmission routes and guarantee rigor in the assignation of clusters indicating ongoing transmission events.

We evaluated the efficiency of MIRU-VNTR in studying the clonal composition of a selection of recurrent cases by analyzing our collection of frozen-stored isolates directly, without subculturing them or purifying DNA for the molecular study. It is common in the clinical context that MTB is cultured from a patient who had a previous TB episode and the isolate from the first episode is available in the strain-collection of the microbiology department. However, it is required to subculture the stored isolate before performing the comparative analysis, which means a great delay. The design we evaluate offers the possibility of analyzing stored isolates directly, in order to rapidly discriminate between reactivation and exogenous reinfection.

For 29 of the 32 selected isolates we obtained data (in at least 10 of the 12 loci studied), which shows the high power of resolution of MIRU-VNTR typing in this challenging setting. In some cases, certain loci failed to be amplified directly from the stored isolates, and they could be amplified from new subcultures; however we have preferred to show only the results obtained from the direct amplification of stored isolates. In the case of the two loci that could not be often amplified (loci 10 and 16), we had previously observed (data not shown) that they were less efficiently amplified even with purified DNA samples. A remarkable feature is that the analysis could be performed even with isolates stored over a long period (study period: 1990–2006) and most of them were successfully amplified.

The 13 patients in our study population yielded 5 cases with some degree of clonal heterogeneity. In three of these cases (patients I, J, K) changes in two or more loci were observed between the recurrent isolates, suggesting an exogenous reinfection with a different strain. These results were confirmed by the clearly different RFLPtypes and spoligotypes after subculturing and analyzing the same isolates. It is worth noting that in two of these three cases, reinfection was associated with a change in the susceptibility patterns, from a susceptible strain to an INH-resistant strain, which is highly relevant for therapy. The patients analyzed in this study were different to those selected in a previous report [5] (33% reinfections) and, again, the rates of reinfection (23%) were higher than expected for a context with a moderate incidence of TB. The good correlation between VNTR-MIRU data and RFLP/spoligotyping indicated that differences in a small number of loci (in our case, two) could identify different strains without requiring standard genotyping analysis. In this sense, a recent study in Shanghai [20] found that differences even in only one tandem repeat in a single MIRU locus enabled the identification of reinfections that were later confirmed by RFLP.

Although this study analyzed recurrent cases to distinguish reinfection from reactivation we found indirectly that the MIRU-VNTR approach succeeded in detecting clonal complexity other than reinfection. We identified another two cases with clonal heterogeneity (patients A and L) but only subtle differences in one locus were detected. One of the cases (L) had only 6 bands in the RFLPtype and it could be a non clonal case [21]. However, these patients could also be likely examples of microevolutions leading to the appearance of clonal variants more than examples of reinfections, although appearance of clonal variants linked to MIRU-VNTR microevolution events has been found to be rather infrequent in other studies (2–6% of the clonal cases)[22]. These recurrent isolates shared identical RFLP and spoligotypes which suggests that MIRU-VNTR has a higher sensitivity for detecting subtle genetic differences, although other studies [14] have shown that MIRU failed to detect subtle differences in RFLPtypes. The appearance of clonal variants in the context of an infection is not usually considered relevant but we report that it could be significant as seen in a case infected by two clonal variants with high genotypic similarity but with different infective behaviours; only one of them was able to infect the CNS whereas the other was restricted to the respiratory site [9]. The potential differential ability to cross the blood-brain barrier between both coinfecting variants is now being analyzed.

The situation of one of the patients whom clonal variants were found (case A) was complex, with a likely combination of microevolution, coinfection and competition. Here, our results could indicate that from an initial strain responsible for the first episode, another clonal variant was originated by genetic drift. The appearance of this second clone could be associated with the acquisition of resistance to pyrazinamide. Both clonal variants coinfected the patient over four years and finally, the original clone competed with the microevolved clone and the infection recovered its initial clonal homogeneity, with only the initial clone infecting the patient. However, it could also be considered that the absence of the second clone in the last specimen was caused by a sampling effect. Anyway, this interesting case shows that MTB infection could be a very dynamic process in clonal terms.

Our sample might be considered too small to enable clonal heterogeneity to be detected, nevertheless we detected a high percentage of these cases which suggests that clonal variability could be detected more frequently than expected. Moreover, the clonal complexity detected in this study reveals only a part of the real situation due to a potential underdetection of clonal heterogeneity caused by i) the fact that we could not obtain information from two loci in many cases, ii) the notion that MIRU 12 has low discriminatory power compared with a recently developed version with 15 loci [23,24], and iii) the potential selection of certain MTB clones that could be present in clinical specimens but that could be counterselected and masked after culture.

## Conclusion

In summary, MIRU-VNTR is an efficient technique for studying the clonal composition of MTB even in challenging laboratory circumstances such as the direct analysis of frozen-stored isolates without subculturing them or purifying DNA. It offers the possibility of rapidly answering clinical requests for identification of exogenous reinfection, reactivation or coinfection during the infection of patients with recurrent TB.

## Methods

### Patients

We analyzed patients from the general population with two or more TB episodes (MTB-positive culture) at least six months apart, during the period 1990–2006. From the 3237 cases with frozen-stored MTB clinical isolates for that period of time, 58 (1.8%) were recurrent cases. From these, we selected 13 cases (all autochthonous Spanish cases) that were representative of a different number of episodes with different intervals between episodes. All but one (urine, case J) corresponded to respiratory samples.

### Samples

The stored frozen samples contained two loopfuls of MTB culture suspended in 1.5 ml of storage medium (7H9 Middlebrook 4.7 g/ml, sucrose 5%, glycerin 2%, OADC 10%). The frozen samples had been stored at -70C from culture until analysis.

### MIRU-VNTR typing

DNA was extracted by sonication in a water-bath sonicator (GenProbe) as described elsewhere [25]. Between 500 and 700μl of the stored culture was sonicated for seven minutes in the presence of 106-micrometers glass beads (SIGMA). DNA was not purified. 5 microliters of the crude extract was used for the typing assays by MIRU-VNTR using the 12-loci format [12]. Amplified products were run in a 2% MS8 agarose gel (Pronadisa, Madrid, Spain) for 16.5 hours at 75V to calculate the number of tandem repeats for each locus.

The potential involvement of laboratory cross-contamination in the misassigment of genotypes was ruled out by comparing the genotypes of all the isolates coprocessed (within a 3-day period) in the laboratory for each of the samples.

### IS6110-RFLP and spoligotyping

For the cases in which MIRUtyping detected genotypic differences between the isolates from the recurrent episodes, additional typing by IS6110-RFLP and spoligotyping was performed. For this purpose, the stored clinical MTB isolates were subcultured in Lowenstein-Jensen slants for three weeks, DNA was purified from the culture and RFLP and spoligotyping were performed following standard procedures [26,27]. Computer-assisted analysis of fingerprints was carried out using Bionumeric 5.1 software.

### Susceptibility test

Susceptibility testing against isoniazid, rifampin, streptomycin, pyrazinamide and ethambutol was performed using the mycobacterial growth indicator SIRE system (Becton Dickinson).

## Authors' contributions

Ana Martín: She has performed all the experimental assays and the MIRUtyping, she has analyzed the results and produced the first version of the MS

Marta Herránz: She has done the RFLP and spoligotyping

María Jesús Ruiz Serrano: She has done all the microbiological procedures

Emilio Bouza: He has revised critically the final version of the MS

Darío García de Viedma*: He has designed the study, supervised all experimental work, analyzed the results, corrected and produced the final version of the MS
